# Progressive systemic inflammation precedes decompensation in compensated cirrhosis

**DOI:** 10.1016/j.jhepr.2024.101231

**Published:** 2024-10-05

**Authors:** Rubén Sánchez-Aldehuelo, Càndid Villanueva, Joan Genescà, Juan Carlos García-Pagán, Elisa Castillo, José Luis Calleja, Carles Aracil, Rafael Bañares, Luis Téllez, Lorena Paule, Rosa María Morillas, María Poca, Beatriz Peñas, Salvador Augustin, Juan G. Abraldes, Edilmar Alvarado-Tapias, Jaume Bosch, Agustín Albillos

**Affiliations:** 1 Department of Gastroenterology and Hepatology, Hospital Universitario Ramón y Cajal, Instituto Ramon y Cajal de Investigación Sanitaria (IRYCIS), Universidad de Alcalá, Madrid, Spain; 2 Centro de Investigación Biomédica en Red de Enfermedades Hepáticas y Digestivas (CIBERehd), Instituto de Salud Carlos III, Madrid, Spain; 3 Department of Gastroenterology, Hospital Santa Creu i Sant Pau, Autonomous University of Barcelona, Department of Medicine, Biomedical Research Institute Sant Pau (IIB Sant Pau), 08025 Barcelona, Spain; 4 Liver Unit, Digestive Diseases Area, Vall d’Hebron University Hospital, Vall d’Hebron Institute of Research (VHIR), Vall d’Hebron Barcelona Hospital Campus, Autonomous University of Barcelona, Barcelona, Spain; 5 Barcelona Hepatic Hemodynamic Laboratory, Liver Unit, Institute of Digestive and Metabolic Diseases, August Pi i Sunyer Institute of Biomedical Research, Hospital Clínic, Health Care Provider of the European Reference Network on Rare Liver Disorders (ERN-RareLiver), Departament de Medicina i Ciències de la Salut, Universitat de Barcelona, Barcelona, Spain; 6 Department of Gastroenterology and Hepatology, Hospital Universitario Puerta de Hierro-Majadahonda, Puerta de Hierro Hospital Research Institute, Universidad Autónoma de Madrid, Madrid, Spain; 7 Department of Gastroenterology and Hepatology, Institute of Biomedical Research, Arnau de Vilanova University Hospital (IRB Lleida), Lleida, Spain; 8 Gastroenterology and Hepatology Department, Hospital Universitario Gregorio Marañón, Instituto de Investigacion Sanitaria Gregorio Marañón (IiSGM), Universidad Complutense de Madrid, Madrid, Spain; 9 Liver Section, Hospital Universitari Germans Trias i Pujol, IGTP, Badalona, Spain; 10 Liver Unit, Division of Gastroenterology, University of Alberta, Edmonton, Canada; 11 Department of Visceral Surgery and Medicine, Inselspital, Bern University Hospital, University of Bern, Switzerland

**Keywords:** cytokine, bacterial translocation, portal hypertension, chronic advanced liver disease, immunity

## Abstract

**Background & Aims:**

Systemic inflammation is a driver of decompensation in cirrhosis with unclear relevance in the compensated stage. We evaluated inflammation and bacterial translocation markers in compensated cirrhosis and their dynamics in relation to the first decompensation.

**Methods:**

This study is nested within the PREDESCI trial, which investigated non-selective beta-blockers for preventing decompensation in compensated cirrhosis and clinically significant portal hypertension (CSPH: hepatic venous pressure gradient ≥10 mmHg). Blood biomarkers were measured at baseline and at 1 and 2 years in patients who remained compensated and had available samples (n = 164). Values of patients with CSPH were split at each time point by decompensation development in the next time interval after sampling. We also included 54 patients with cirrhosis and subclinical portal hypertension (PH) and 35 controls. We assessed markers of inflammation (interleukin-6 [IL-6], tumor necrosis factor-alpha, von Willebrand factor [vWF], C-reactive protein), macrophage activation (CD14, CD163), intestinal barrier integrity (fatty acid-binding protein [FABP], haptoglobin), and bacterial translocation (lipopolysaccharide [LPS]).

**Results:**

IL-6, CD163, and vWF were higher (*p <*0.01) at baseline in patients with cirrhosis and CSPH compared to those with subclinical PH and controls. IL-6 increased (*p <*0.05) at 1 year in patients with CSPH, with a greater rise in those who developed decompensation. CD163 was higher (*p <*0.01) in patients who decompensated at baseline and 1 and 2 years. FABP was elevated (*p <*0.01) in patients with CSPH compared to subclinical PH and controls at baseline and 1 year, while haptoglobin was lower (*p <*0.01). LPS was higher (*p <*0.01) in patients with CSPH than in those with subclinical PH and controls and increased at 1 year regardless of decompensation development.

**Conclusions:**

Inflammation and bacterial products are present in the systemic circulation in patients with compensated cirrhosis and CSPH. Progressive inflammation precedes the first decompensation.

**Impact and implications:**

Systemic inflammation drives cirrhosis progression during the decompensated stage, but its role in the compensated stage is unclear. We evaluated biomarkers of systemic inflammation, intestinal barrier integrity and bacterial translocation in patients with compensated cirrhosis and their dynamics in relation to the first decompensation. We demonstrate that low-grade inflammation and bacterial products are present in the systemic circulation in compensated cirrhosis, provided clinically significant portal hypertension has developed. We also show that worsening of systemic inflammation precedes the development of first clinical decompensation.

## Highlights

• Systemic inflammation drives cirrhosis progression during decompensation, but its role in the compensated stage is unclear.
• Low-grade inflammation and bacterial products are present in the systemic circulation in compensated cirrhosis with CSPH.
• Worsening of systemic inflammation precedes the development of first clinical decompensation.

## Graphical abstract

**Figure:**
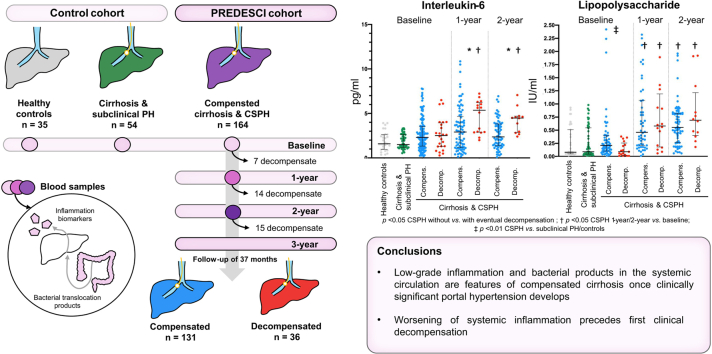

## Introduction

Portal hypertension (PH) is a hallmark of cirrhosis and a key factor in the progression from the compensated to the decompensated stage. Portal pressure, estimated by the hepatic venous pressure gradient (HVPG), should reach 10 mmHg (*i.e*., clinically significant portal hypertension [CSPH]) for hyperdynamic circulation to develop, placing patients at risk of decompensation.^1^^,^^2^ Low-grade systemic inflammation (SI) drives decompensated cirrhosis by worsening systemic circulatory dysfunction.[3], [4], [5] As cirrhosis decompensates, SI intensifies leading to further deterioration and progression to acute-on-chronic liver failure.^6^

SI in cirrhosis is primarily caused by the translocation of enteric pathogen-associated molecular patterns into the bloodstream due to gut barrier dysfunction. This dysfunction is distinctively present in decompensated cirrhosis, with severity paralleling disease progression in both experimental models and patients.[7], [8], [9] Notably, the anti-inflammatory effects of non-selective beta-blockers (NSBBs) are more pronounced in Child-Pugh C patients with more severe SI.^5^ However, evidence on SI and gut barrier damage in compensated cirrhosis is limited and inconsistent.^8^^,^^10^^,^^11^

We hypothesize that, similar to hyperdynamic circulation, SI does not occur in compensated cirrhosis until CSPH is present and that worsening SI precedes the first decompensation.

This study evaluated patients with compensated cirrhosis, both with and without CSPH, to characterize SI and its dynamics in relation to the onset of the first decompensation.

## Patients and methods

### Patients

This study included patients from three cohorts:

Compensated cirrhosis with CSPH. This group comprised patients from a registered serum sample collection (C.0005353, Biobank Registry of Instituto de Salud Carlos III, Madrid) linked to a randomized trial evaluating NSBBs for preventing decompensation in compensated cirrhosis with CSPH (PREDESCI trial; NCT01059396).^12^^,^^13^ Inclusion criteria required cirrhosis with HVPG ≥10 mmHg and no prior decompensation or high-risk esophageal varices. Exclusions included prior decompensation, HVPG <10 mmHg, hepatocellular carcinoma, bilirubin >3 mg/dl, creatinine >2 mg/dl, international normalized ratio >1.7, or platelet count <30,000/μl.

Compensated cirrhosis with subclinical PH. This cohort consisted of patients from Hospital Universitario Ramón y Cajal with subclinical PH (HVPG 6-9 mmHg) who underwent HVPG measurement due to clinical indications, and who were selected based on similar inclusion/exclusion criteria and cirrhosis etiology distribution as the PREDESCI cohort. None were on lactulose or rifaximin.

Healthy controls. Age and sex-matched healthy controls were also included for comparison.

Samples from the latter two cohorts were provided by the BioBank Hospital Ramón y Cajal-IRYCIS (National Registry of Biobanks B.0000678) and processed according to procedures with Ethical and Scientific Committees approval CTAT table).

### Study design

In the cirrhosis with CSPH cohort, biomarkers and HVPG were measured at baseline, and at 1 and 2 years in patients who remained compensated. In the subclinical PH and control cohorts, biomarkers were measured only at baseline.

### Sample analysis

Blood samples were collected and stored at -80 °C until analysis. Different biomarkers were studied to assess SI (interleukin-6 [IL-6], tumor necrosis factor-alpha [TNF-α], von Willebrand factor [vWF], C-reactive protein [CRP]), macrophage activation (CD14, CD163), intestinal barrier dysfunction (intestinal fatty acid-binding protein [FABP], haptoglobin) and bacterial translocation (lipopolysaccharide-binding protein [LBP], lipopolysaccharide [LPS]). IL-6 and TNF-α were determined by high-sensitivity ELISA from Quantikine (R&D Systems, Minneapolis, MN, USA), CRP by high-sensitivity immunoturbidimetry (Alinity, Abbott, Germany), vWF, CD14, CD163, LBP, and haptoglobin by ELISA from DuoSet (R&D Systems), FABP by ELISA from Hycult (Biotech, Uden, The Netherlands), and LPS by a Limulus Amebocyte Lysate assay (Lonza, Hayward, CA, USA).

### Statistical analysis

Statistical analyses were performed using Stata/SE 14.0 (StataCorp, College Station, TX, USA). All tests were two-tailed, with a *p* value <0.05 considered significant. Categorical variables are reported as proportions, and continuous variables as mean ± SD or median (IQR), depending on data distribution assessed by the Shapiro-Wilk test. The Mann-Whitney, Kruskal-Wallis, and Wilcoxon signed-rank tests were used for comparing continuous variables, with Bonferroni correction applied for multiple comparisons in the Kruskal-Wallis test.

## Results

### Patients and follow-up

In the present study, we included those patients from the PREDESCI cohort with a blood sample available at inclusion. In these patients, samples available at 1 and 2 years were also analyzed. Due to violations in the management protocol, samples were not available for 37 patients at inclusion, and in another 49, and 12 patients at 1 and 2 years, respectively (Fig. S1). Therefore, biomarkers were measured in samples from 164 patients at inclusion, 108 at 1 year, and 82 at 2 years. We included 54 patients in the cohort with cirrhosis and subclinical PH and 35 healthy controls.

Table S1 shows the characteristics of the patients. The main etiology of cirrhosis in patients with CSPH as well as in those with subclinical PH was hepatitis C virus, followed by alcohol. During a median follow-up of 37 months, 36 of the 164 patients with CSPH (21.9%) decompensated, mainly because of ascites (17.7%) (Table S2). The median time to decompensation was 23.5 months, with seven decompensations occurring in the first year (19.4%), 14 between years 1 and 2 (38.9%), and 15 after year 2 (41.6%).

### Peripheral blood biomarkers

Results for the biomarkers assessed across the different cohorts are summarized in Table 1.

**Table 1:** Blood biomarkers in healthy controls and in patients with compensated cirrhosis with subclinical PH and CSPH.

	Baseline			1 year†			2 year‡		
	Median	IQR	*p* values	Median	IQR	*p* intra	Median	IQR	*p* intra
IL-6 (pg/ml)									
Controls (n = 35)	1.66	1.01-2.64	**<0.05**^,^#			**<0.05**			0.12
Cirrhosis with subclinical PH (n = 54)	1.5	1.12-2.66							
Cirrhosis with CSPH (n = 164)	2.32	1.31-3.56		3.03	1.39-5.25		2.71	1.53-4.15	
TNF (ng/ml)									
Controls (n = 35)	6.41	5.3-7.44	0.52			0.59			0.25
Cirrhosis with subclinical PH	6.46	5.61-7.8							
Cirrhosis with CSPH	6.1	4.1-8.4		6.83	4.77-8.79		6.58	3.96-9.88	
vWF (ng/ml)									
Controls	2.95	1.46-5.13	**<0.01**∗^,^#			0.58			0.43
Cirrhosis with subclinical PH	3.39	1.87-5.37							
Cirrhosis with CSPH	6.0	3.61-9.81		4.79	2.78-9.18		4.25	2.59-9.96	
C-reactive protein (mg/dl)									
Controls	0.59	0.35-1.21	0.06			0.6			0.46
Cirrhosis with subclinical PH	0.85	0.6-1.08							
Cirrhosis with CSPH	0.52	0.19-1.17		0.55	0.18-1.43		0.5	0.2-1.41	
CD163 (ng/ml)									
Controls (n = 35)	258.74	210.7- 362.4	**<0.01**∗^,^#			0.5			0.6
Cirrhosis with subclinical PH	252.85	205.6-305.55							
Cirrhosis with CSPH	857.72	605.5-1,117		820.35	524.2-1,087.1		787.67	534.37-1,174.81	
CD14 (mg/ml)									
Controls	1.23	0.84-1.48	0.2			0.77			0.4
Cirrhosis with subclinical PH	1.49	0.98-1.88							
Cirrhosis with CSPH	1.28	1.07-1.6		1.27	1-1.58		1.22	1.02-1.51	
LBP (μg/ml)									
Controls	6.2	4.1-6.9	0.66			0.89			0.25
Cirrhosis with subclinical PH	5.9	5.4-7.1							
Cirrhosis with CSPH	6.05	4.65-7.39		6.31	5.26-7.62		6.75	5.05-8.26	
LPS (IU/ml)									
Controls	0.05	0.03-0.08	**<0.01**∗^,^#			**<0.01**			**<0.01**
Cirrhosis with subclinical PH	0.09	0.05-0.12							
Cirrhosis with CSPH	0.20	0.11-0.36		0.54	0.21-1.07		0.58	0.28-1.14	
FABP (ng/ml)									
Controls	0.31	0.19- 0.50	**<0.01**∗^,^#			0.95			0.18
Cirrhosis with subclinical PH	0.46	0.31-0.53							
Cirrhosis with CSPH	0.72	0.52-1.04		0.73	0.419-1.01		0.88	0.56-1.52	
Haptoglobin (mg/L)									
Controls	721.5	425.9-988.5	**<0.01**∗^,^#			0.22			0.45
Cirrhosis with subclinical PH	750.2	589-830							
Cirrhosis with CSPH	213.4	72.94-349.2		224.3	98.6-366.7		227.4	76.94-381.8	

*p* values: controls *vs*. cirrhosis with subclinical PH *vs.* cirrhosis with CSPH (Kruskal-Wallis test with Bonferroni correction).

*p* intra: baseline vs. 1-year and 2-year, respectively, in cirrhosis with CSPH (Wilcoxon signed-rank test).

*p* values <0.05 indicated in bold.

CSPH, clinically significant portal hypertension; FABP, fatty acid binding protein; LBP, lipopolysaccharide-binding protein; LPS, lipopolysaccharide; PH, portal hypertension; vWF, von Willebrand factor.

∗ Controls *vs*. cirrhosis with CSPH.

# Cirrhosis with subclinical PH *vs*. cirrhosis with CSPH.

† Cirrhosis with CSPH (n = 108).

‡ Cirrhosis with CSPH (n = 82).

#### Systemic inflammation and macrophage activation markers

Serum levels of IL-6, CD163, and vWF were greater (*p <*0.01) at baseline in patients with CSPH compared to those with subclinical PH and controls. There were no significant differences in these biomarkers between the subclinical PH cohort and controls. Within the CSPH group, biomarker levels were similar in patients with and without small varices (Table S3) and across the different etiologies of cirrhosis (Table S4).

**Fig. 1:**
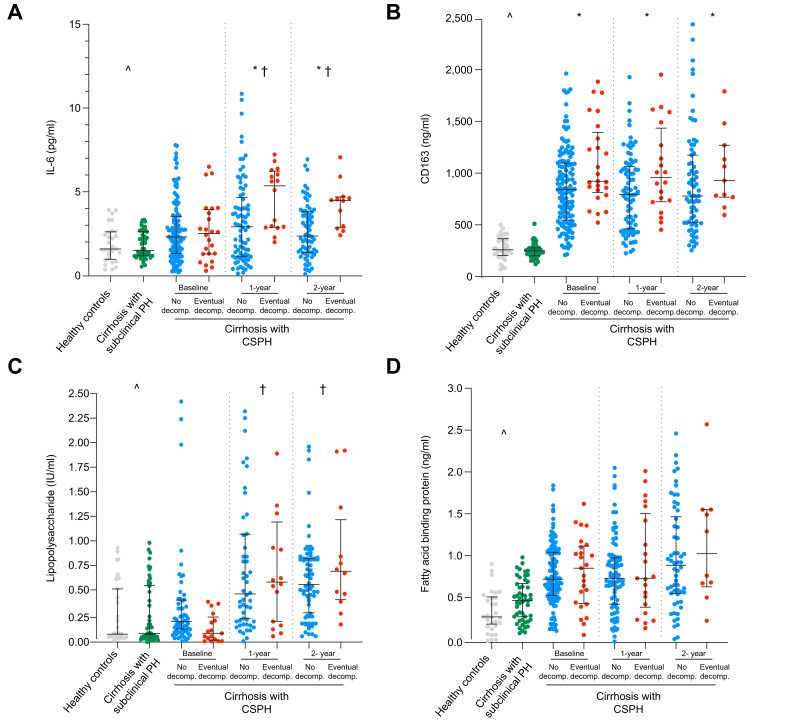
Blood biomarkers in healthy controls and in patients with compensated cirrhosis with clinically significant and subclinical PH. Points represent individual data and horizontal lines p25, p50 and p75. Values of patients with CSPH have been split at each time point by the development of decompensation in the next time interval after sampling. Statistical analysis: Kruskal-Wallis test to compare continuous variables among groups at baseline, Bonferroni correction test when *p <*0.05 and Wilcoxon signed-rank test to compare continuous variables between the groups. (A) Interleukin-6: Levels of significance: ^ˆ^*p =* 0.03; ∗1-year *p =* 0.01, ∗2-year *p =* 0.03; ^†^1-year *p =* 0.03, ^†^2-year *p =* 0.04. (B) CD163. Levels of significance: ^ˆ^*p =* 0.01; ∗baseline *p =* 0.01, ∗1-year *p =* 0.01, ∗2-year *p =* 0.04. (C) Lipopolysaccharide. Levels of significance: ^ˆ^*p =* 0.01; ^†^1-year *p =* 0.01; ^†^2-year *p =* 0.01. (D) Fatty acid-binding protein. Levels of significance: ^ˆ^*p =* 0.01. ^ˆ^Subclinical PH *vs*. CSPH baseline. ∗CSPH without *vs.* with eventual decompensation. ^†^CSPH 1-year/2-year *vs.* baseline. CSPH, clinically significant portal hypertension; PH, portal hypertension.

Serum IL-6 significantly increased from 2.32 pg/ml to 3.03 pg/ml (*p =* 0.02) at 1 year in patients with cirrhosis and CSPH. This rise was largely driven by patients who eventually decompensated, whose IL-6 levels increased from 2.52 pg/ml to 5.36 pg/ml (+112.7%, *p =* 0.03), compared to those who did not decompensate (+25.4%, *p =* 0.07) (Fig. 1). CD163 levels were similar at baseline and 1 and 2 years in patients with CSPH, but were significantly (*p <*0.05) higher in patients who eventually decompensated compared to those who did not at all time points. Similar results were observed when only patients with ascites were considered (Table S5). No correlation was found between biomarker levels and bacterial infections during follow-up (Table S6).

#### Intestinal barrier dysfunction and bacterial translocation markers

FABP was higher (*p <*0.01) in patients with CSPH compared to controls at baseline, while haptoglobin levels were lower. The subclinical PH group showed similar FABP and haptoglobin levels to the control group but differed significantly (*p <*0.001) from the CSPH group. No differences in these biomarkers were observed in the CSPH group at follow-up or in relation to eventual decompensation (Table 1, Fig. 1).

LPS levels were higher (*p <*0.01) in patients with CSPH than in those with subclinical PH and controls (Table 1). LPS significantly increased (*p <*0.01) at 1 and 2 years in both patients who eventually decompensated and those who did not (Fig. 1). The absolute variation (delta) did not differ between the two groups.

#### Correlations between biomarkers

As shown in Fig. S2, we found significant (*p <*0.05), though weak, correlations in the cohorts of patients with cirrhosis, both with and without CSPH, between markers of inflammation (IL-6) and markers of macrophage activation (CD163, r = 0.31; CD14, r = 0.26), intestinal barrier integrity (FABP, r = 0.28), and HVPG (r = 0.21). LPS was weakly but significantly (*p <*0.05) correlated with FABP (r = 0.20), CD14 (r = 0.23), and HVPG (r = 0.26). Instead, CD163 was significantly (*p <*0.05) correlated with other markers of gut barrier integrity, such as haptoglobin (r = -0.43). We found significant (*p <*0.05) associations between vWF and CD163 (r = 0.34), CD14 (r = 0.36), and haptoglobin (r = -0.43), but not with any of the other biomarkers studied.

We observed significant (*p <*0.05), though weak, associations between the absolute changes from baseline to 1 year in IL-6 with TNF (r = 0.32), vWF (r = 0.25), CD163 (r = 0.3), CD14 (r = 0.26), CRP (r = 0.30) and LBP (r = 0.27), as well as those of CD163 with CD14 (r = 0.31), CRP (r = 0.21) and LBP (r = 0.26) (Fig. S3).

### NSBBs and HVPG response at 1 year

No differences in biomarker values were observed between the groups of patients on placebo or NSBBs at 1 year (Table S7). There were also no differences in biomarkers according to whether the patients achieved a HVPG reduction ≥10% of baseline at 1 year (data not shown).

## Discussion

In the present study, we explored whether SI is present in patients with compensated cirrhosis with CSPH and whether changes in the grade of SI predict a first clinical decompensation. We measured different biomarkers in two cohorts of patients with compensated cirrhosis, one with CSPH and the other with subclinical PH, as well as in a cohort of healthy controls. Our results show that in patients with compensated cirrhosis 1) low-grade inflammation and bacterial products are present in the systemic circulation once CSPH develops, and 2) SI worsens during follow-up before decompensation occurs.

Compared to patients with subclinical PH and controls, those with CSPH in our study exhibited low-grade SI, characterized by elevated serum levels of IL-6, a SI biomarker, CD163, a macrophage activation marker, and vWF, an endothelial activation marker. Unlike other studies,^13^^,^^14^ serum TNF-α and CRP were not increased, possibly due to the early stage of cirrhosis and cohort homogeneity, as well as differences in cirrhosis etiology. Remarkably, SI severity was higher at baseline and worsened at 1 year in patients who eventually decompensated. Patients who eventually decompensated had higher levels of IL-6 and CD163 at baseline, which further increased at 1 year compared to those who did not decompensate. This pattern of SI has also been described in patients with acute decompensation who eventually progress to acute-on-chronic liver failure.^6^

Our study provides some insights regarding the potential trigger of SI. FABP and haptoglobin, markers of gut barrier integrity, were increased and decreased, respectively, in patients with CSPH compared to those with subclinical PH. Patients with CSPH also showed elevated levels of the pathogen-associated molecular pattern LPS, which worsened during follow-up, and correlated at baseline with FABP and CD14. It is tempting to speculate that monocyte activation driven by enteric LPS might contribute to SI. This is supported by the significant correlations of the macrophage activation markers, CD163 and CD14, with IL-6 and haptoglobin. The absolute changes over 1 year between IL-6, CD163, and LBP further reinforce this idea. Simbrunner *et al.* also reported that bacterial translocation is not limited to patients with decompensated cirrhosis but may already be present in the compensated stage.^15^ Finally, we acknowledge that the strength of the data supporting our hypothesis is relatively weak, due to modest coefficients of the associations and the lack of correlation between LPS and inflammation markers. However, it is important to consider that the episodic nature of endotoxemia in cirrhosis and the short half-life of endotoxin complicate the interpretation of a single determination to reflect a trend over time, especially in terms of its correlation with biomarkers resulting from immune system stimulation.

Firstly, the predominant etiology of cirrhosis was hepatitis C, which may not reflect the current cirrhotic population, potentially affecting the inflammatory pathways. However, our study did not reveal trends supporting this hypothesis. Additionally, biomarkers were measured only at baseline and fixed yearly intervals, lacking temporal alignment with decompensation or bacterial infections, which limits detailed analysis. Moreover, the baseline, 1-year, and especially 2-year cohorts were not fully overlapping due to missing serum samples, possibly reducing the statistical power of comparisons. In addition, the missing data raises questions about whether the information provided by available samples accurately reflects that of the whole patient population. Despite these limitations, our study offers valuable insights and may prompt further research into the role of SI in decompensation in compensated cirrhosis.

In conclusion, our data suggest that low-grade SI and bacterial products in the systemic circulation are features of compensated cirrhosis once CSPH has developed. This implies that the elevated portal pressure above a certain threshold plays a crucial role in the occurrence of SI, similar to its impact on hyperdynamic circulation. In this context, worsening of SI precedes first clinical decompensation.

## Abbreviations

CRP, C-reactive protein; CSPH, clinically significant portal hypertension; FABP, fatty acid-binding protein; HVPG, hepatic venous pressure gradient; IL-6, interleukin-6; LBP, lipopolysaccharide-binding protein; LPS, lipopolysaccharide; MELD, model for end-stage liver disease; NSBBs, non-selective beta-blockers; PH, portal hypertension; SI, systemic inflammation; vWF, Von Willebrand factor.

## Financial support

Supported by grants from the 10.13039/501100004837Ministerio de Ciencia e Innovación and 10.13039/501100004587Instituto de Salud Carlos III (PI20/01302 to A.A., PI21/01995 to E.A.T.). R.S.A. and E.A.T. are recipients of grants from the Ministerio de Ciencia e Innovación and Instituto de Salud Carlos III (CM20/00020, JR20/00047). 10.13039/501100015755Centro de Investigación Biomédica en Red en Enfermedades Hepáticas y Digestivas (CIBEREHD) is funded by the Instituto de Salud Carlos III with grants cofinanced by the European Development Regional Fund “A way to achieve Europe” (EDRF).

## Authors’ contribution

All authors have made substantial contributions and satisfy the criteria for authorship: conception and design; analysis and interpretation of the data; drafting of the article; critical revision of the article for important intellectual content; final approval of the article. Conceptualization: RSA, AA. Data curation: RSA, CV, JG, JCGP, EC, JLC, CA, RB, LT, LP, RMM, MP, BP, SA, JGA, EAT, JB, AA. Formal analysis: RSA, AA. Funding acquisition: AA. Investigation: RSA, AA. Methodology: RSA, AA. Supervision: AA. Drafting of the manuscript: RSA, AA. Critical revision of the manuscript for important intellectual content: RSA, CV, JG, JCGP, EC, JLC, CA, RB, LT, LP, RMM, MP, BP, SA, JGA, EAT, JB, AA.

## Data availability statement

The data that support the findings of this study are available from the corresponding author upon reasonable request.

## Conflicts of interest

The authors have declared that no personal or financial competing interests exist. Please refer to the accompanying ICMJE disclosure forms for further details.
