# Supplementary material for: Progressive systemic inflammation precedes decompensation in compensated cirrhosis
Source: JHEP Rep. 2024 Oct 5;7(2):101231. doi: 10.1016/j.jhepr.2024.101231 (PMC11754518; doi:10.1016/j.jhepr.2024.101231)
Supplement: Multimedia component 1 [file mmc1.pdf]

Progressive systemic inflammation precedes decompensation in compensated cirrhosis

Rubén Sánchez Aldehuelo, Càndid Villanueva, Joan Genescà, Juan Carlos García-Pagán, Elisa Castillo, José Luis Calleja, Carles Aracil, Rafael Bañares , Luis Téllez , Lorena Paule, Rosa María Morillas, María Poca, Beatriz Peñas, Salvador Augustin, Juan Abraldes, Edilmar Alvarado-Tapias, Jaume Bosch, Agustín Albillos

Table of contents

Fig. S1 ……………………………………………………………………………………2

Fig. S2 ……………………………………………………………………………………3

Fig. S3 ……………………………………………………………………………………4

Table S1 …………………………………………………………………………………5

Table S2 …………………………………………………………………………………6

Table S3 …………………………………………………………………………………7

Table S4 …………………………………………………………………………………8

Table S5 …………………………………………………………………………………9

Table S6 ………………………………………………………………………………..12

Table S7 ………………………………………………………………………………..15

Fig. S1. Flowchart of the patients included in each cohort.

The chart shows the number of patients with cirrhosis and clinically significant portal hypertension with samples analyzed at each timepoint

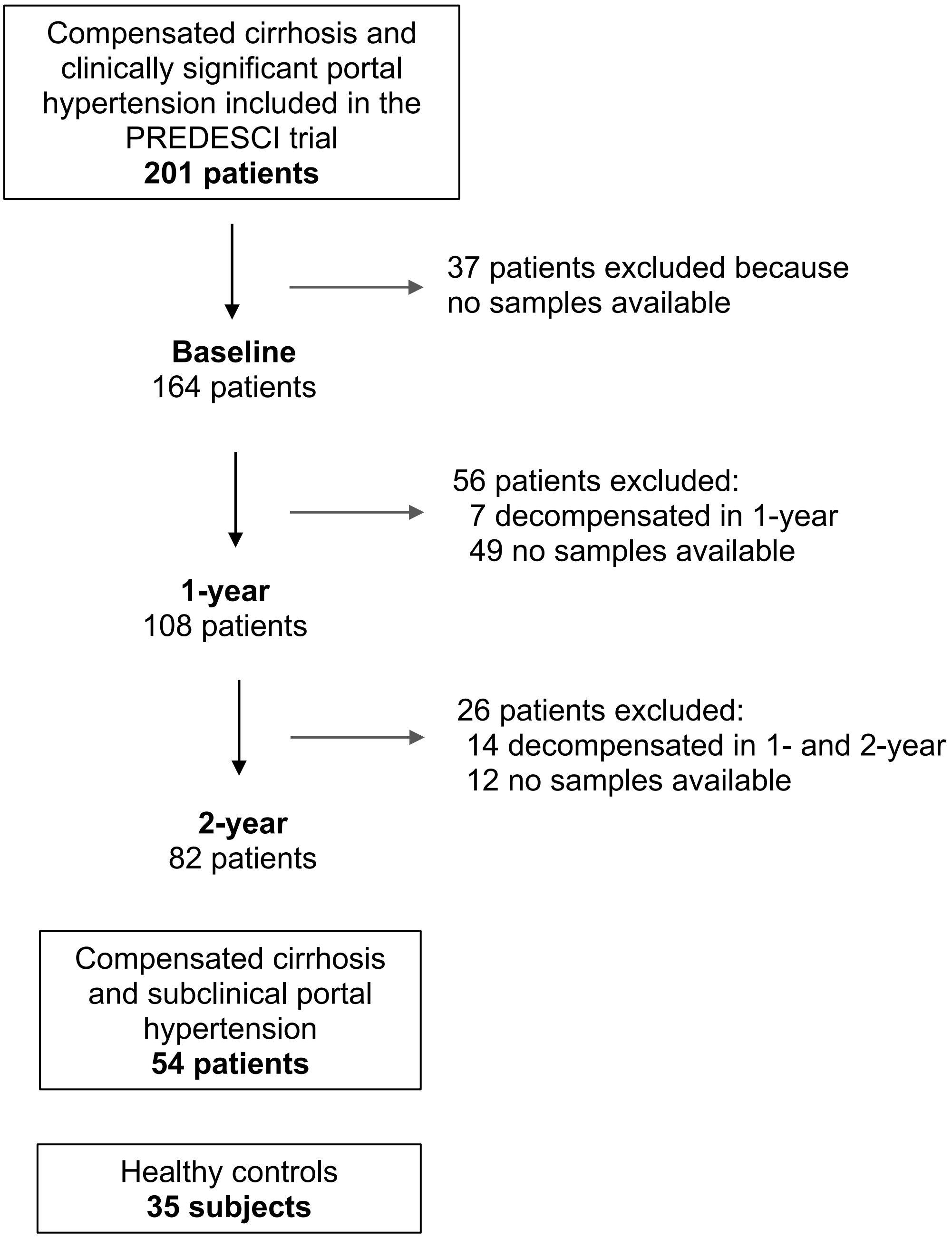

Fig. S2. Correlations of baseline values of serum biomarkers and hepatic venous pressure gradient in patients with compensated cirrhosis with clinically significant portal hypertension (n=164) and with subclinical portal hypertension (n=54)

Statistical analysis: Spearman's correlation coefficients were calculated to assess the association between continuous variables.

Legend: * p<0.05, ** p<0.01, *** p<0.001

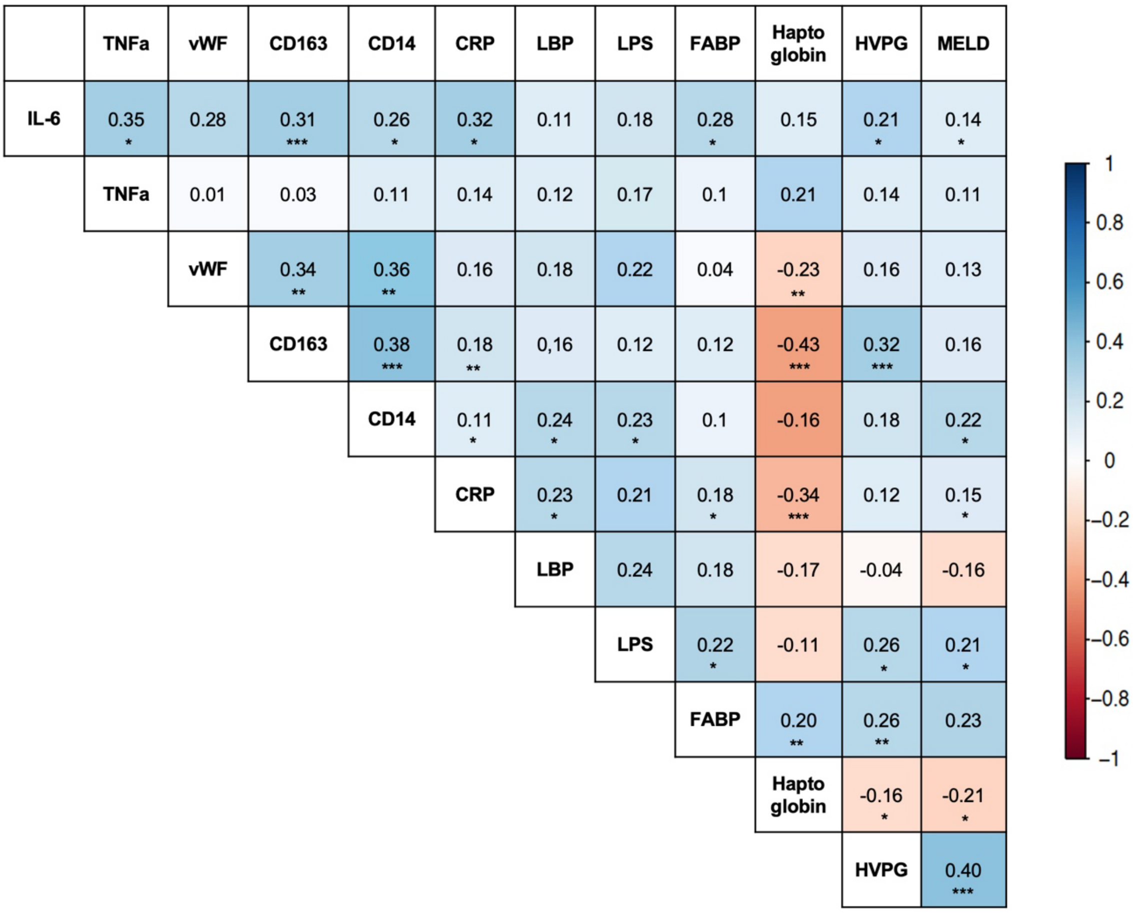

Fig. S3. Correlations of changes in the values of serum biomarkers at baseline and at 1 year in patients with compensated cirrhosis with clinically significant portal hypertension in patients with paired measurements (n=108)

Statistical analysis: Spearman's correlation coefficients were calculated to assess the association between continuous variables.

Legend: * p<0.05, ** p<0.01, *** p<0.001

	TNFa	vWF	CD163	CD14	CRP	LBP	LPS	FABP	Hapto globin
IL-6	0.32 **	0.25 *	0.30 **	0.26 *	0.30 **	0.27 *	0.06	0.1	0.02
	TNFa	0.03	0.13	0.1	0.24 *	0.01	0.16	0.24 *	0.03
		vWF	0.1	0.06	0.07	0.13	0.09	-0.14	-0.17
			CD163	0.31 **	0.21 *	0.26 *	0.15	0.08	-0.12
				CD14	0.14	0.57 ***	-0.02	0.1	0.1
					CRP	0.42 ***	0.19	0.07	-0.20
						LBP	0.1	0.08	-0.1
							LPS	0.05	-0.18
								FABP	-0.02

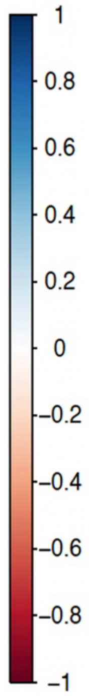

Table S1. Baseline characteristics of patients and healthy controls

	**Compensated cirrhosis and subclinical portal hypertension (n=54)**	**Compensated cirrhosis and clinically significant portal hypertension (n=164)**	**Healthy controls n=35**
**Male,** n (%)	11 (55.0)	101 (61.5)	20 (57.1)
**Age** (years), mean ± SD	62.4 ± 8.1	58.3 ± 10	60.6 ± 9.2
**Etiology,** n (%)			
**Alcohol**	8 (15.0)	25 (15.2)	
**HCV**	32 (59.9)	92 (56.0)	
**Alcohol and HCV**	8 (15.0)	12 (73.1)	
**MASLD**	6 (10.1)	11 (6.7)	
**Others**		24 (14.6)	
**Child-Pugh,** n (%)			
**A**	54 (100)	134 (81.8)	
**B**		30 (18.2)	
**MELD score,** mean ± SD	6.0 ± 0.2	6.6 ± 0.2	
**Esophageal varices,** n (%)	54 (100)	74 (45.2)	
**None**		90 (54.8)	
**Small**			
**Hepatic venous pressure gradient** (mmHg), mean±SD	7.4 ± 2	14.8 ± 4	

HCV, hepatitis C virus. MASLD, metabolic associated steatotic liver disease

Table S2. Events on follow-up of patients with compensated cirrhosis and clinically significant portal hypertension

	**Compensated cirrhosis with clinically significant portal hypertension (n=164)**
**Median follow-up** (months)	36
**Decompensation,** n (%)	36 (21.9)
**Ascites**	29 (17.6)
**Variceal bleeding**	7 (4.2)
**Hepatic encephalopathy**	9 (5.4)
**Bacterial infection** n (%)	34 (20.7)
**Time to event (months),** median (p25-p75)	
**Ascites**	23.5 (13.6-34.4)
**Variceal bleeding**	21.8 (11.5-29)
**Hepatic encephalopathy**	23.5 (19.2-34.6)
**Liver transplant, n (%)**	4 (2)
**Death, n (%)**	19 (11.6)

Table S3. Blood biomarkers in patients with compensated cirrhosis with clinically significant portal hypertension according to the absence (n=79) or presence (n=85) of small varices

		**Median**	**IQR**	**p**
**IL-6 (pg/mL)**	No varices	2.57	1.31-3.56	ns
	Small varices	2.29	1.3-3.32	
**TNF (ng/mL)**	No varices	6.27	4.5-8.87	ns
	Small varices	5.98	3.64-7.55	
**vWF (ng/mL)**	No varices	5.46	3.68-9.51	ns
	Small varices	6.1	3.53-10	
**C reactive protein (mg/dL)**	No varices	0.39	0.14-1.04	ns
	Small varices	0.33	0.16-0.81	
**CD163 (ng/mL)**	No varices	886.17	534.94-1088.29	ns
	Small varices	841.16	610.86-1196.71	
**CD14 (mg/mL)**	No varices	1.34	1.1-1.68	ns
	Small varices	1.21	1.03-1.55	
**LBP (µg/mL)**	No varices	6.18	4.58-7.15	ns
	Small varices	5.92	4.88-7.39	
**LPS (IU/mL)**	No varices	0.24	0.07-0.39	ns
	Small varices	0.18	0.08-0.31	
**FABP (ng/mL)**	No varices	0.63	0.48-0.93	ns
	Small varices	0.75	0.56-1.06	
**Haptoglobin (mg/L)**	No varices	236.354	86.20-392.75	ns
	Small varices	191.41	74.54-342.19	

IQR: interquartilic range (p25-p75);

FABP: intestinal fatty acid binding protein; LBP, lipopolysaccharide binding protein; LPS, lipopolysaccharide; vWF: von Willebrand factor

Table S4. Blood biomarkers in patients with compensated cirrhosis with subclinical and with clinically significant portal hypertension according to the etiology of cirrhosis

	Alcohol n=34	HCV n=112	Alcohol and HCV n=20	MAFLD n=16	Others n=24	p
**IL-6 (pg/mL)**	2.32 (0.79-3.87)	2.26 (1.43-3.25)	2.75 (1.31-5.47)	2.98 (0.86-4.04)	2 .01 (0.86-2.83)	ns
**TNF (ng/mL)**	5.28 (3.22-7.43)	6.07 (3.84-8.81)	5.69 (4.51.9.1)	4.94 (4.3-6.16)	6.94 (4.94-8.04)	ns
**vWF (ng/mL)**	5.62 (4.08-6.04)	6.21 (3.61-9.53)	6.87 (6.39-11.79)	7.79 (2.9-12.2)	5.15 (2.15-7.145)	ns
**C reactive protein (mg/dL)**	0.63 (0.45-1.04)	0.58 (0.22-0.81)	0.65 (0.26-0.89)	0.78 (0.09-1.6)	0.61 (0.37-1.25)	ns
**CD163 (ng/mL)**	703.54 (536.5-977.9)	898.9 (720.2-1106.7)	874.6 (681-928)	781.45 (495.2-991.72)	727.13 (621.1-894.7)	ns
**CD14 (mg/mL)**	1.1 (0.97-1.22)	1.3 (1.1-1.58)	1.43 (1.1-1.8)	1.35 (1.15-1.67)	1.28 (1.04-1.8)	ns
**LBP (µg/mL)**	5.5 (5.29-6.29)	6.44 (4.9-7.6)	6.38 (5.04-7.86)	5.77 (3.23-6.57)	5.87 (4.06-6.7)	ns
**LPS (IU/mL)**	0.21 (0.13-0.34)	0.18 (0.07-0.36)	0.14 (0.04-0.28)	0.2 (0.13-0.36)	0.15 (0.08-0.76)	ns
**FABP (ng/mL)**	0.72 (0.43-1.06)	0.7 (0.56-1.04)	0.96 (0.72-1.06)	0.52 (0.39-1.04)	0.69 (0.45-1)	ns
**Haptoglobin (mg/L)**	271.8 (148.8-532.9)	195.79 (44.5-322.2)	196.25 (64.5-218.1)	254.8 (264.9-382.2)	198.76 (117.2-245.1)	ns

Data shown as median and interquartilic range (p25-p75)

ns, not significant;

FABP: intestinal fatty acid binding protein; HCV, hepatitis C virus; LBP, lipopolysaccharide binding protein; LPS, lipopolysaccharide; MAFLD, metabolic associated fatty liver disease; vWF: von Willebrand factor

Table S5. Blood biomarkers in patients with compensated cirrhosis with clinically significant portal hypertension without (n=130) and with (n=29) ascites on follow-up

		Baseline				1-year			2-year			
		**Median**	**IQR**	**p**		**Median**	**IQR**	**p intra**		**Median**	**IQR**	**p intra**
**IL-6 (pg/mL)**	No ascites	2.33	1.34-3.57	0.51	No ascites	2.98	1.17-4.78	**0.04**	No ascites	2.71	1.43-4	0.19
	Ascites	2.36	1.06-3.55		Ascites	5.36	2.87-6.04	**0.07**	Ascites	3.28	1.9-4.6	1
**TNF (ng/mL)**	No ascites	6.12	4.11-8.39	0.16	No ascites	6.85	4.43-8.47	0.84	No ascites	6.56	4.03-10.2	0.31
	Ascites	5.47	3.62-6.18		Ascites	5.59	4-6.74	0.38	Ascites	7.3	3.33-8.92	0.61
**vWF (ng/mL)**	No ascites	6.01	3.68-10	0.66	No ascites	4.81	2.74-8.79	0.39	No ascites	4.62	2.46-7.92	0.59
	Ascites	5.64	3.21-9.26		Ascites	4.37	3.06-9.35	0.72	Ascites	4.07	3.12-8.51	0.72
**C reactive protein (mg/dL)**	No ascites	0.38	0.15-0.88	0.58	No ascites	0.45	0.18-1.11	**<0.01**	No ascites	0.47	0.19-1.41	**<0.01**
	Ascites	0.51	0.24-0.84		Ascites	0.58	0.3-1.36	0.92	Ascites	0.66	0.38-1.38	0.18

**CD163 (ng/mL)**	No ascites	841.16	555.45-1105.41	**0.01**	No ascites	809.48	468.381-1087.1	0.84	No ascites	779.54	521.43-1174.81	0.64
	Ascites	921.65	857.72-1334.84		Ascites	904.84	720.21-1141.06	0.81	Ascites	929.13	783.13-1133.13	0.13
**CD14 (mg/mL)**	No ascites	1.28	1.06-1.58	0.85	No ascites	1.28	1-1.57	0.94	No ascites	1.26	1.02-1.55	0.96
	Ascites	1.24	1.08-1.7		Ascites	1.16	1.06-1.62	0.41	Ascites	1.17	1.03-1.32	0.87
**LBP (µg/mL)**	No ascites	6.03	4.65-7.25	0.58	No ascites	6.27	5.25-7.72	0.84	No ascites	6.71	5.05-8.23	0.09
	Ascites	6.06	5.05-8.41		Ascites	6.71	5.59-7.5	0.51	Ascites	6.91	5.58-8.26	0.17
**LPS (IU/mL)**	No ascites	0.2	0.1-0.39	0.08	No ascites	0.48	0.21-1.01	**<0.01**	No ascites	0.50	0.28-1.12	0.9
	Ascites	0.11	0.04-0.29		Ascites	0.58	0.17-0.93	**0.03**	Ascites	0.53	0.14-0.98	0.6
**FABP (ng/mL)**	No ascites	0.72	0.53-1.04	0.85	No ascites	0.73	0.42-0.98	0.9	No ascites	0.88	0.56-1.47	**<0.001**
	Ascites	0.8	0.42-1.13		Ascites	0.73	0.42-1.42	0.6	Ascites	0.98	0.59-1.55	0.12
**Haptoglobin (mg/L)**	No ascites	215.61	86.2-365.23	0.3	No ascites	224.33	98.57-370.71	0.16	No ascites	228.65	83.51-384.43	0.4

	Ascites	147.34	33.52-327.35		Ascites	224.62	111.49-345.93	1	Ascites	120.54	25.32-288.82	0.5

p: controls vs. cirrhosis with subclinical PH vs. cirrhosis with CSPH. **p intra:** baseline vs. 1 year and 2 year, respectively, in cirrhosis with CSPH p-values <0.05 indicated in bold. ns, not significant

IQR: interquartilic range (p25-p75)

FABP: intestinal fatty acid binding protein; LBP, lipopolysaccharide binding protein; LPS, lipopolysaccharide; vWF: von Willebrand factor

Table S6. Blood biomarkers in patients with compensated cirrhosis with clinically significant portal hypertension without (n=130) and with (n=34) bacterial infection on follow-up

		Baseline				1-year				2 year		
		**Median**	**IQR**	**p**		**Median**	**IQR**	**p intra**		**Median**	**IQR**	**p intra**
**IL-6 (pg/mL)**	No infection n=130	2.34	1.32-3.6	0.58	No infection n=81	2.93	1.15-4.78	**0.04**	No infection n=64	2.86	1.44-3.88	0.26
	Infection n=34	2.01	1.15-3.2		Infection n=24	3.67	2.08-5.8	0.13	Infection n=19	3.5	1.53-4.95	0.6
**TNF (ng/mL)**	No infection	5.87	3.93-7.74	0.58	No infection	6.28	4.43-8.12	0.34	No infection	6.37	3.88-9	0.27
	Infection	6.3	4.1-8.91		Infection	6.28	2.97-8.47	0.35	Infection	8.94	5.38-16.76	0.53
**vWF (ng/mL)**	No infection	6	3.61-10.03	0.83	No infection	4.79	2.71-8.79	0.56	No infection	4.76	2.54-8.03	0.51
	Infection	6.5	3.66-8.68		Infection	4.78	2.82-9.35	0.94	Infection	4.83	2.90-9.88	0.82
**C reactive protein**	No infection	0.38	.15-0.84	0.47	No infection	0.45	0.17-1.05	**<0.01**	No infection	0.46	0.2-1.4	**<0.01**

**(mg/dL)**	Infection	0.36	0.24-1.05		Infection	0.81	0.2-1.55	0.38	Infection	0.68	0.15-2.2	0.16
**CD163 (ng/mL)**	No infection	856.4	597-1117.22	0.84	No infection	814.69	518.68-1062.81	0.61	No infection	776.01	534.38-1024.14	0.96
	Infection	912.14	612.68-1112.64		Infection	902.43	577.3-1267.27	0.07	Infection	1174.82	532.87-1790.62	0.07
**CD14 (mg/mL)**	No infection	1.29	1.09-1.6	0.15	No infection	1.27	0.98-1.5	0.13	No infection	1.26	1.02-1.6	0.44
	Infection	1.08	1-1.58		Infection	1.26	1.09-1.73	**<0.01**	Infection	1.17	1-1.42	0.11
**LBP (µg/mL)**	No infection	5.95	4.64-7-1	0.09	No infection	6.06	4.97-7.17	0.6	No infection	6.64	5-8.54	0.36
	Infection	6.57	4.74-8.87		Infection	7.65	6.06-9.48	0.43	Infection	7.36	5.59-8.26	0.59
**LPS (IU/mL)**	No infection 59	0.2	0.09-0.4	0.47	No infection 53	0.47	0.18-1.06	**<0.01**	No infection	0.49	0.19-1.16	**<0.01**
	Infection 18	0.175	0.07-0.29		Infection 15	0.62	0.33-1.28	**0.01**	Infection	0.60	0.35-1.31	**<0.01**
**FABP (ng/mL)**	No infection 123	0.7	0.49-1.04	0.15	No infection 79	0.7	0.42-0.99	0.74	No infection 51	0.86	0.55-1.35	**<0.01**

	Infection	0.85	0.63-1.16		Infection	0.76	0.47-1.06	0.68	Infection	0.92	0.63-1.73	0.05
**Haptoglobin (mg/L)**	No infection	212.25	71.35-349.43	0.91	No infection	221.92	92.31-364.45	0.29	No infection	221.21	60.76-395.1	0.53
	Infection	216.463	78.05-342.19		Infection	243.39	143.16-389.96	0.57	Infection	227.39	76.93-382.31	0.82

p: controls vs. cirrhosis with subclinical PH vs. cirrhosis with CSPH.

p intra: baseline vs. 1 year and 2 year, respectively, in cirrhosis with CSPH

p-values <0.05 indicated in bold.

IQR: interquartilic range (p25-p75);

FABP: intestinal fatty acid binding protein; LBP, lipopolysaccharide binding protein; LPS, lipopolysaccharide; vWF: von Willebrand factor

Table S7. Blood biomarkers in patients with compensated cirrhosis with clinically significant portal hypertension on placebo (n=84) or NSBB (n=80) at baseline and at 1 year

		Baseline				1-year		
		**Median**	**IQR**	**p**		**Median**	**IQR**	**p**
**IL-6 (pg/mL)**	Placebo	2.49	1.51-3.12	0.82	Placebo	2.84	1.39-4.19	0.24
	NSBB	2.21	1.16-4.04		NSBB (n= 50)	3.27	1.33-5.81	
**TNF (ng/mL)**	Placebo	5.92	4.51-8.04	0.25	Placebo	6.52	4.77-8.25	0.62
	NSBB	5.6	3.6-8.22		NSBB	6.17	4.23-7.96	
**vWF (ng/mL)**	Placebo	6.11	3.7-9.82	0.52	Placebo	3.94	2.62-8.82	0.44
	NSBB	5.65	2.55-9.43		NSBB	4.97	3.51-9.38	
**C reactive protein (mg/dL)**	Placebo	0.4	0.15-0.9	0.76	Placebo	0.41	0.18-1.26	0.21
	NSBB	0.38	0.17-0.82		NSBB	0.79	0.33-1.34	
**CD163 (ng/mL)**	Placebo	878.6	681.17-1117.41	0.23	Placebo	785.71	521.71	0.36
	NSBB	828.33	523.28-1115.38		NSBB	878.57	524.19-1118.74	
	Placebo	1.28	1.08-1.6	0.54	Placebo	1.25	0.99-1.58	0.96

**CD14 (mg/mL)**	NSBB	1.27	1.05-1.58		NSBB	1.3	1.06-1.57	
**LBP (µg/mL)**	Placebo	6.29	5.04-7.6	0.32	Placebo	6.83	5.52-7.62	0.17
	NSBB	5.92	4.62-7.2		NSBB	6.09	4.72-7.64	
**LPS (IU/mL)**	Placebo	0.19	0.07-0.35	0.58	Placebo	0.45	0.18-1.07	0.51
	NSBB	0.19	0.1-0.31		NSBB	0.6	0.23-1.06	
**FABP (ng/mL)**	Placebo	0.73	0.57-1.03	0.51	Placebo	0.75	0.56-1	0.3
	NSBB	0.68	0.47-1.06		NSBB	0.69	0.36-0.96	
**Haptoglobin (mg/L)**	Placebo	202.32	61.66	0.55	Placebo	222.16	96.43-355.64	0.66
	NSBB	213	72.94-349.18		NSBB	245.23	100.71-382.19	

IQR: interquartilic range (p25-p75); NSBB, non-selective beta-blockers

FABP: intestinal fatty acid binding protein; LBP, lipopolysaccharide binding protein;

LPS, lipopolysaccharide; vWF: von Willebrand factor

JHEP Reports
CTAT methods

Tables for a "Complete, Transparent, Accurate and Timely account" (CTAT) are now mandatory for all revised submissions. The aim is to enhance the reproducibility of methods.

- Only include the parts relevant to your study
- Refer to the CTAT in the main text as 'Supplementary CTAT Table'
- Do not add subheadings
- Add as many rows as needed to include all information
- Only include one item per row

If the CTAT form is not relevant to your study, please outline the reasons why:

1.1 Antibodies

Name	Citation	Supplier	Cat no.	Clone no.

1.2 Cell lines

Name	Citation	Supplier	Cat no.	Passage no.	Authentication test method

1.3 Organisms

Name	Citation	Supplier	Strain	Sex	Age	Overall n number

1.4 Sequence based reagents

Name	Sequence	Supplier

1.5 Biological samples

Description	Source	Identifier
BioBank Hospital Ramón y Cajal-IRYCIS		National Registry of Biobanks B.0000678

1.6 Deposited data

Name of repository	Identifier	Link

1.7 Software

Software name	Manufacturer	Version

1.8 Other (*e.g*. drugs, proteins, vectors etc.)

1.9 Please provide the details of the corresponding methods author for the manuscript:

2.0 Please confirm for randomised controlled trials all versions of the clinical protocol are included in the submission. These will be published online as supplementary information.

